# Narrative strategies in live-streaming commerce: host storytelling, immersion, and impulse buying

**DOI:** 10.3389/fpsyg.2026.1746938

**Published:** 2026-01-21

**Authors:** Mengdi Wang, Wenjing Zhang, Yanxia Li

**Affiliations:** 1 Harbin Institute of Technology Weihai, Weihai, China; 2 School of Management, Northeastern University at Qinhuangdao, Qinhuangdao, China; 3 School of Economics and Management, Yanshan University, Qinhuangdao, China

**Keywords:** immersion, impulse buying, live-streaming host, mixed-methods, narrative transportation theory

## Abstract

Live-streaming hosts play a pivotal role in e-commerce, yet their narration strategies remain underexplored. This study develops a framework to examine how host narration strategies influence consumers’ impulsive buying behavior. The exploratory phase involved textual analysis of host narration, which informed the conceptual model grounded in Narrative Transportation Theory. In the confirmatory phase, 398 user responses were examined using structural equation modeling (SEM) to test the path model and fuzzy set qualitative comparative analysis (fsQCA) to identify sufficient configurations of influencing factors. Results show that emotional persuasion, narrative presence, and immersion are consistently associated with impulsive buying, while interactive engagement, time pressure, and accuracy have context-specific effects. These findings emphasize the need to adapt live-streaming strategies flexibly to match consumer characteristics and situational contexts, offering actionable insights for enhancing consumer engagement and purchase behavior in live-streaming commerce.

## Introduction

Live-streaming e-commerce has become a dominant force in digital retail, offering consumers real-time interaction, product demonstrations, and a highly immersive shopping experience. Unlike traditional e-commerce, live shopping creates a sense of urgency and social presence that can trigger spontaneous purchasing decisions (Wongkitrungrueng and Assarut, 2020). Impulse buying, characterized by unplanned and emotionally driven decisions, is particularly prominent in such fast-paced and emotionally engaging environments (Lin et al., 2023).

While prior research has explored consumer behavior in live-streaming contexts, much of it has focused on environmental cues or consumer traits, placing less emphasis on the live-streaming host—the central figure who shapes the consumer experience. Hosts play a dual role: they demonstrate products and cultivate emotional connections through real-time interaction, storytelling, and expressive communication. Previous studies have highlighted external attributes such as trustworthiness, expertise, and charisma (Guo et al., 2022), as well as social features like comments, likes, and interactive dialogues that foster consumer participation (Ma et al., 2022). Similarly, some studies have found that common interaction methods such as likes and follows, which are prevalent on social platforms, can also effectively stimulate consumer spending (Gao et al., 2021; Luo et al., 2024). Recent review and meta-analytic studies further suggest that research on live-streaming commerce and impulse buying has expanded rapidly, yet existing findings remain fragmented across diverse drivers, mechanisms, and outcomes. In particular, prior syntheses highlight that much of the literature adopts a variable-centered perspective, emphasizing isolated cues or individual factors, while offering limited insight into how multiple host-related cues jointly shape consumers’ immersive experiences and impulsive responses (Lee et al., 2025; Xu et al., 2025). Against this backdrop, a more integrative perspective that examines hosts’ narrative strategies as a coherent stimulus system remains underexplored.

However, existing research primarily emphasizes “signifiers,” focusing on external attributes such as host credibility and gender, while paying limited attention to the “signified,” namely the underlying verbal strategies and content that shape consumer engagement. As the live commerce industry becomes saturated with similar formats, repetitive narratives risk inducing consumer fatigue. Thus, optimizing the narrative strategies of hosts has become essential to sustain engagement and enhance the shopping experience.

To address this gap, the present study adopts a narrative perspective to examine how host storytelling shapes consumer immersion and impulsive behavior through emotional appeal and narrative coherence. Drawing on narrative transportation theory, we explore how consumers become psychologically transported into the live-streaming context and how this immersive experience influences their purchase intentions.

This study employs a mixed-method design. In Study 1, qualitative content analysis identifies key narrative features used by hosts. Based on these findings, Study 2 develops a hypothesis model and tests it using both Partial Least Squares Structural Equation Modeling (PLS-SEM) and Fuzzy-Set Qualitative Comparative Analysis (fsQCA). By integrating qualitative themes with quantitative validation, this research offers a comprehensive understanding of how narrative features influence consumer engagement and provides new insights into the psychological mechanisms of live-stream commerce.

## Background literature

### Narrative transportation theory

Narrative transportation theory explains how individuals become mentally immersed in a story world, influencing their attitudes and behaviors (Green and Brock, 2000). When transported, individuals exhibit cognitive attention, emotional engagement, and detachment from reality. This state reduces resistance to persuasion and increases content acceptance (Green, 2006).

Four main factors influence narrative transportation: (1) Narrative content, where coherence and vividness—logical flow, causal links, and descriptive richness—sustain attention and emotional resonance (Bruner, 1986); (2) Narrator characteristics, such as credibility and emotional delivery, which foster empathy and deepen engagement (Green and Clark, 2013); (3) Audience traits, including cognitive involvement and emotional sensitivity, affecting how stories are received (Slater and Rouner, 2002); and (4) Communication context, where high-interaction, immersive settings enhance presence and narrative effects (Gerrig, 1993).

In the live-streaming environment, hosts function as both storytellers and product advocates. They use emotionally expressive language and narrative techniques to contextualize products within relatable scenarios, increasing specificity and realism. These narratives foster audience immersion and connection. (Liu et al., 2023). Real-time interactivity—Q&A, demos, and flash sales—enhances situational relevance and urgency, boosting emotional involvement and narrative immersion. This weakens rational evaluation and promotes emotion-driven behaviors, such as impulse purchases (Kang et al., 2021). Thus, host narration strategies are vital tools for narrative transportation, shaping consumer emotions, perceptions, and purchase decisions.

### Flow theory and immersion

Flow theory describes a psychological state in which individuals experience intense focus, emotional involvement, and a sense of intrinsic enjoyment while engaging in an activity (Csikszentmihalyi, 1990). While flow emphasizes internal experience, immersion expands this by incorporating external stimuli that enhance presence and psychological involvement. Immersion involves cognitive focus, emotional engagement, and situational presence, resulting from both internal responses and external cues (Agarwal and Karahanna, 2000; Jennett et al., 2008).

In live-streaming shopping, immersion is a key mechanism for understanding user engagement and decision-making. Hosts use emotional appeals, vivid narratives, and interactive tools—such as real-time responses and flash sales—to trigger immersion. These elements foster resonance, focus, and presence, drawing viewers into the content and making them more receptive to persuasion (Yim et al., 2017). Thus, flow and immersion together offer a strong framework to explain how consumers become psychologically absorbed in live-streaming contexts and why this absorption is associated with impulsive purchases.

## Research design

This study adopts a mixed-methods approach to overcome the limitations of relying solely on a single research method. By combining the in-depth insights of qualitative analysis with the reliability of quantitative methods, the study provides a more comprehensive and in-depth understanding of the research phenomena.

The qualitative phase focuses on exploring the key characteristics of live-streaming hosts’ narration strategies to build a conceptual foundation for the study. This phase employs a systematic thematic analysis, designed to capture intricate details of the phenomenon under investigation. The qualitative findings offer a nuanced understanding of the constructs that inform hypothesis development and guide the subsequent quantitative investigation.

In the quantitative phase, we first use SEM to examine the relationships between the key factors identified in the qualitative phase and consumer immersion and impulsive buying behavior. Next, we employ fsQCA to explore the configurational effects between variables, providing insights into the combinations of factors that influence impulsive buying behavior. By integrating the advantages of these two methodologies, we gain a comprehensive and in-depth understanding of the characteristics and effects of lice-streaming host narrative strategies.

## Exploratory study (study 1)

### Research objective

The research objective of this study is the narrative strategies of shopping live-streaming hosts, exploring the key features through a combination of different methods. By analyzing live-streaming content, this study attempts to explore which host narration strategies can better influence consumers’ perceptions and behavioral intentions. The research results can provide assistance and reference for optimizing sales effectiveness in live streaming rooms.

### Data collection

In Study 1, we conducted a qualitative study focusing on shopping live-streaming content on Taobao Live. Taobao Live is one of the largest and most mature live-streaming commerce platforms in China, integrating real-time video streaming with e-commerce transactions. It has developed a highly institutionalized live-streaming ecosystem in which hosts routinely rely on narration, real-time interaction, and persuasive communication to promote products. As such, Taobao Live provides a representative and information-rich context for examining live-streaming host narration strategies.

Based on this context, we recorded 10 1-h live-streaming sessions conducted by the top 50 Taobao shopping hosts between August 1, 2023, and September 1, 2023. These sessions captured how hosts explained products and interacted with viewers in real time. The recorded content was transcribed into Chinese, after which thematic analysis was conducted to extract key narrative characteristics. Theoretical saturation was reached after transcribing the sixth live-streaming session, as no new significant themes emerged from the data thereafter.

### Data analysis methodology

We conducted a thematic analysis on the transcribed content to extract meaningful insights. Using a hierarchical coding framework in MAXQDA, we iteratively identified recurring patterns and themes that reflected the hosts’ narrative styles. The coding process involved team discussions to ensure consistency and validity in identifying key themes. To ensure coding reliability, two researchers independently reviewed and coded the transcripts based on the initial coding scheme. The coders then compared their coding results and discussed discrepancies through regular meetings until consensus was reached. The coding framework was iteratively refined during this process to ensure consistency and reliability in identifying key themes.

### Findings and discussion

The thematic analysis identified five primary characteristics of live-streaming hosts’ narratives: emotional persuasion, narrative presence, interactive engagement, time pressure, and accuracy and specificity. Hosts use emotionally charged language to evoke excitement or urgency in consumers, often by highlighting limited availability or exclusive deals (emotional persuasion). Storytelling techniques enhance the sense of immediacy and relevance. In turn, they provide consumers with a greater sense of control and a stronger subjective experience derived from how the experience is presented (narrative presence). At the same time, active interaction with viewers through real-time responses, personalized greetings, and lighthearted exchanges fosters a sense of cohesion and trust (interactive engagement). Time-limited offers are emphasized to encourage impulsive decision-making (time pressure), while providing precise and credible product information enhances consumer confidence (accuracy).

These identified characteristics suggest that live-streaming hosts employ a multifaceted narration strategy, combining emotional appeal, storytelling, and credible information delivery to influence consumer behavior. These strategies align with theoretical perspectives on persuasion and decision-making, which posits that both affective and cognitive elements are critical in shaping consumer actions. Additionally, the findings offer a foundation for developing a comprehensive model to explore the causal relationships between narrative characteristics and impulsive purchasing, which will be further examined in Study 2.

## Confirmatory study (study 2)

### Development of hypotheses

Emotional persuasion in live-streaming shopping refers to hosts’ use of energetic and passionate language to captivate audiences and elicit positive emotional responses. Such emotionally engaging language helps create an inviting and comfortable atmosphere, encouraging viewers to become more emotionally involved in the live-streaming experience (Labrecque, 2014). The host’s enthusiastic and attractive speech serves as an emotional cue, guiding the audience to focus less on cognitive processing and more on their emotional reactions, which aligns with the information processing described by flow theory (Schramm and Hartmann, 2008).

Consumers in shopping live-streaming tend to have more emotional responses while watching the host (compared to rational analytical responses), and a greater amount of emotion can trigger a stronger sense of immersion, further driving consumer participation and experience. In addition, the host’s persuasive language further triggers consumers’ emotional involvement, enhances the audience’s psychological investment, and adds to the immersive experience for consumers (Reinikainen et al., 2020). Therefore, we hypothesize that:

*H1*: Emotional persuasion by the host positively influences viewer immersion

Narrative presence in live-streaming refers to the host’s use of storytelling to create immediacy and engagement. Rather than listing product features, hosts construct vivid, relatable scenarios that emotionally connect with viewers. This fosters a sense of immersion, allowing audiences to mentally simulate product use. According to narrative immersion theory, the deeper individuals feel transported into a story, the less critically they evaluate its content. This process increases empathy and emotional resonance (Green and Clark, 2013). This vicarious experience can influence real-life behavior, as viewers unconsciously adopt characters’ actions.

Narrative content also elicits emotional responses, strengthening psychological connections with the message. As emotional engagement rises, viewers rely less on analytical processing and more on affective responses, making the host’s message more persuasive (Green and Brock, 2000; Slater and Rouner, 2002). This shift enhances immersion in the live-streaming environment and promotes acceptance of the host’s explanation. Thus, we hypothesize that:

*H2:* Narrative presence by the host positively influences viewer immersion

Interactive engagement refers to the host’s real-time interaction with viewers, such as responding to comments and answering questions. These interactive behaviors enhance the audience’s sense of participation and presence (Chen and Lin, 2018). This fosters a feeling of genuine connection, making viewers feel socially involved.

According to the social presence theory, when consumers feel “I am really with others,” they are more likely to immerse themselves in this virtual environment and have a deep experience (Lin and Lee, 2024). Real-time responsiveness deepens shared emotional experiences, strengthening the sense of presence and community. This sense of interactivity increases psychological investment, as viewers feel like active participants rather than passive observers. Moreover, interactive engagement reduces emotional distance between host and viewers, enhancing perceived intimacy and meaning (Huang et al., 2024). Such emotional closeness fosters deeper engagement and promotes immersion. Therefore, we hypothesize that:

*H3*: Interactive engagement by the host positively influences viewer immersion

Live-streaming hosts often use time pressure tactics, such as countdowns and limited-time deals, to create urgency and encourage quicker decision-making. These tactics evoke psychological pressure, compelling viewers to focus on the live event and act quickly.

From a cognitive psychology perspective, time pressure increases cognitive load. While excessive load may hinder decision-making, moderate pressure can enhance attention and focus. In live shopping, perceived urgency prompts viewers to concentrate more on the host’s actions, boosting engagement (Brannon and Brock, 2001).

Emotionally, time pressure can trigger excitement or anxiety, further intensifying immersion. The fear of missing out heightens emotional arousal and creates a sense of urgency, drawing viewers deeper into the shopping context (Haynes, 2009). Every moment in the live stream feels more critical, enhancing both focus and emotional connection. Thus, time-limited promotions are not only sales tactics but also psychological triggers that enhance immersion through both cognitive and emotional pathways. Therefore, we hypothesize that:

*H4*: Time pressure created by the host positively influences viewer immersion

Product information accuracy refers to the host’s delivery of precise, detailed, and truthful product information. This includes descriptions of specifications, features, and usage scenarios. This accuracy fosters trust, which is a key factor in promoting viewer immersion in live-streaming (Kim et al., 2008).

Accurate information reduces cognitive uncertainty and skepticism, helping consumers feel more secure in their purchase decisions. This trust promotes deeper psychological engagement and emotional investment in the experience (Gefen et al., 2003).

Moreover, accuracy enhances cognitive involvement. Clear and reliable product details help viewers process information more effectively, increasing focus and attention to the host’s content (Jiang and Benbasat, 2007). This deepened engagement reinforces immersion during the live-streaming. In addition, information accuracy can trigger positive emotions such as confidence, satisfaction, and reassurance. These emotions further strengthen the immersive experience. When viewers trust the content, they are not only more focused but also more emotionally aligned with the experience. Therefore, we hypothesize that:

*H5*: The accuracy of product information positively influences viewer immersion

Immersion occurs when consumers become deeply engaged in a live-stream shopping session, losing awareness of time and surroundings. It has been associated with heightened emotional involvement and reduced cognitive control, similar to experiences in immersive media like virtual reality (Yim et al., 2017).

Studies show immersion influences consumer behavior across contexts, enhancing engagement (Yang et al., 2021) and purchase intention (Li et al., 2022), and spending through increased perceived control (Song et al., 2019). In this state, consumers become more emotionally driven, lowering resistance to persuasive cues. Impulsive buying, defined as a sudden, unplanned urge to purchase, is more likely when emotional arousal is high. Immersion intensifies this emotional susceptibility, making consumers more responsive to time-limited offers or enthusiastic recommendations from hosts. Therefore, we hypothesize that:

*H6*: Immersion positively influences impulsive buying behavior.

**Figure 1:**
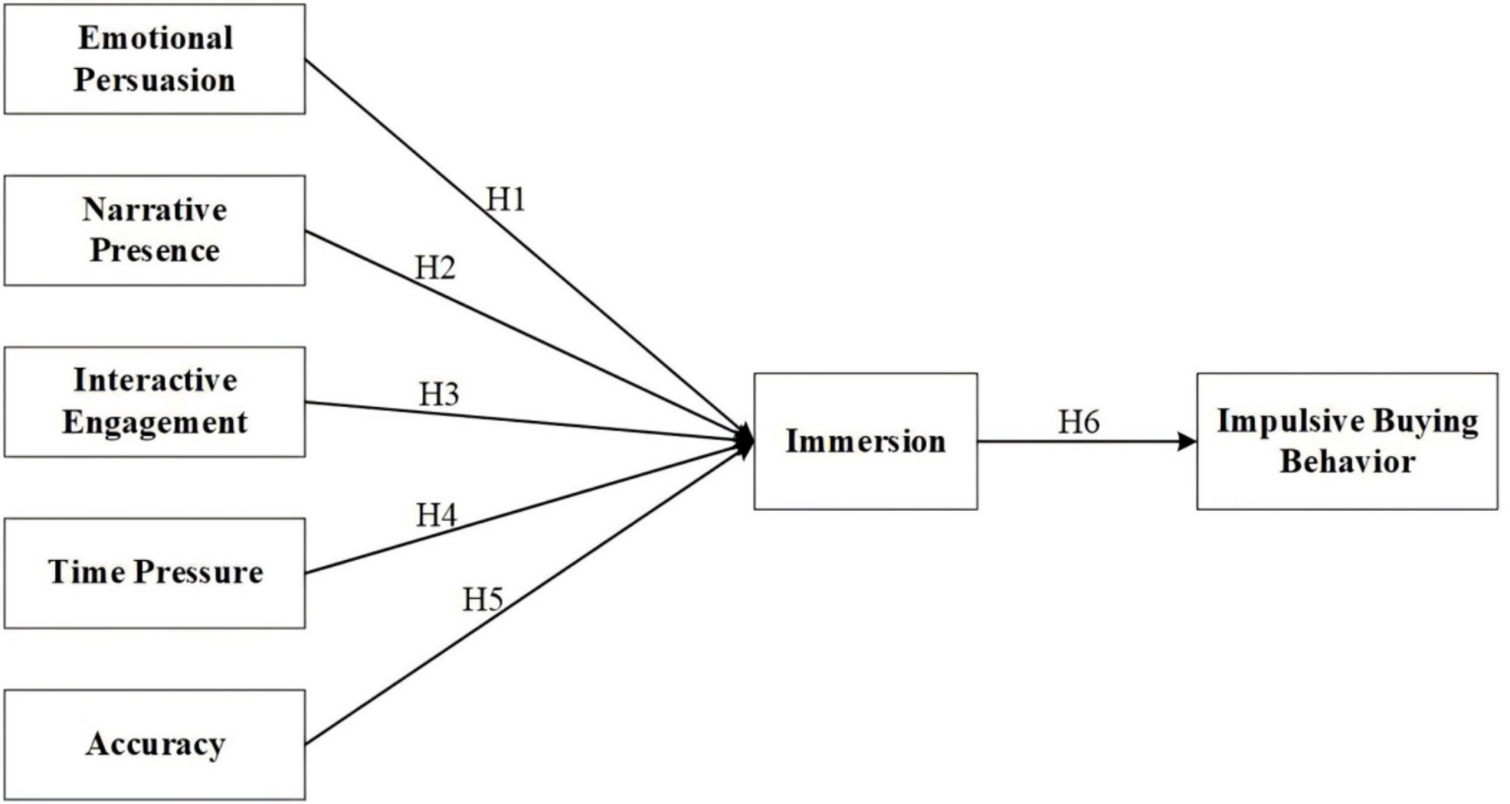
Hypothesized model.

The hypothetical model of this article can be found in Figure 1.

### Research methodology

#### Data collection and sample

To empirically test how the explanations of live-streaming hosts affect consumers’ impulse buying behavior, this study conducted a questionnaire survey in China, where live-streaming e-commerce has been widely adopted and consumer participation is high. The survey was distributed via Credamo, a professional online survey platform widely used in academic research in China, between June and July 2024. Credamo provides access to a large and diverse participant pool and implements multiple data quality control mechanisms, including account-level identity verification, IP restrictions, and response monitoring. While individual identifying information is not disclosed to researchers, these procedures help ensure that responses are provided by real participants rather than automated or duplicate accounts. All participants were informed at the beginning of the survey that their responses would be collected anonymously and used solely for academic research purposes. A brief introduction outlining the study context was provided prior to the questionnaire. Participants were also prompted to recall their recent live-streaming shopping experiences to enhance response consistency and reduce potential recall bias.

A total of 410 questionnaires were initially collected. Prior to the main survey, a pilot test with approximately 50 participants was conducted to assess item clarity and preliminary measurement reliability. As no substantive revisions were required, these responses were retained and included in the final dataset. Invalid responses were excluded based on predefined criteria, including excessively short completion times, patterned or invariant responses, and failed attention-check items. After applying these screening procedures, 398 valid questionnaires were retained for subsequent analysis. Table 1 provides an overview of the demographic characteristics of the sample. The distribution of participants is consistent with the general audience profile of live-streaming commerce, with a higher proportion of young female consumers (KPMG, 2020).

**Table 1:** Demographic details.

Demographic measures	Items	Count	Percent
Gender	Male	124	31%
	Female	274	69%
Age	0–20	8	2%
	21–30	161	41%
	31–40	200	50%
	41–50	20	5%
	51–60	9	2%
	>60	0	0%
Education	Senior high school	8	2%
	Undergraduate	299	75%
	Master	91	23%

#### Measures

The questionnaire items were adapted from established scales and slightly reworded to fit the context of live-streaming commerce. Emotional persuasion items were adapted from Walters et al. (2010), narrative presence from Busselle and Bilandzic (2009), interactive engagement from Yoo et al. (2010), time pressure from Gärling et al. (2015), accuracy from Wixom and Todd (2005), immersion from Gong et al.(2024), and impulsive buying from Fu and Hsu (2023). All items were modified to reflect the live-streaming shopping scenario while retaining their original conceptual meanings. The full list of measurement items is provided in Appendix A.

To ensure the consistency and understanding of the target sample, a two-step translation process (i.e., back-translation) was conducted by two authors, with all items translated twice: first from English to Chinese, and then another person translated from Chinese back to English. By comparing and analyzing the items in different language versions, the final version of the questionnaire items was modified and organized to ensure conceptual consistency. All items were measured using a 5-point Likert scale, ranging from 1 (“strongly disagree”) to 5 (“strongly agree”).

##### Common method variance assessment

Two statistical analyses were performed to control the common method bias (CMB). Harman’s single-factor test was performed first. It was found that the first factor accounted for 33.005% of the total variance—less than the threshold of 50% of the overall variance (Podsakoff et al., 2003), suggesting that CMB was not a serious concern.

In addition, as suggested by Liang et al. (2007), we added a common method factor that links all the items of the principal constructs in the PLS model and calculated each indicator’s variances substantively accounted for by the principal construct and by the common method factor, respectively (Dong and Wang, 2018). Then we examined the average variance explained by substantive constructs and the common method factor and the results in Table 2 indicated that the ratio of the substantive variance to method variance was about 134:1, meaning the indicators’ substantive variances were substantially higher than their method variances. Additionally, each substantive factor loading was significant, while the majority of the common method factor loadings were insignificant. These results suggested that the effect of CMB was minimal.

**Table 2:** Assessment of common method variance using a common method factor approach.

Construct	Indicator	Substantive factor loading (R1)	R1^2^	Method factor loading (R2)	R2^2^
EP	EP1	0.792**	0.628	−0.055	0.003
	EP2	0.703**	0.494	0.168**	0.028
	EP3	0.789**	0.622	−0.059	0.003
	EP4	0.789**	0.623	−0.039	0.002
NP	NP1	0.879**	0.772	0.049	0.002
	NP2	0.850**	0.722	−0.060	0.004
	NP3	0.850**	0.722	0.009	0.000
IE	IE1	0.847**	0.717	−0.130**	0.017
	IE2	0.773**	0.598	0.032	0.001
	IE3	0.802**	0.643	0.081	0.007
	IE4	0.827**	0.684	0.023	0.001
TP	TP1	0.916**	0.838	0.007	0.000
	TP2	0.899**	0.809	−0.017	0.000
	TP3	0.933**	0.870	0.009	0.000
Acc	Acc1	0.829**	0.688	0.065	0.004
	Acc2	0.771**	0.595	−0.007	0.000
	Acc3	0.833**	0.694	−0.059	0.003
Imm	Imm1	0.744**	0.554	0.171*	0.029
	Imm2	0.765**	0.585	−0.061	0.004
	Imm3	0.754**	0.569	−0.004	0.000
	Imm4	0.727**	0.529	−0.113	0.013
IB	IB1	0.869**	0.755	0.002	0.000
	IB2	0.833**	0.694	−0.032	0.001
	IB3	0.848**	0.719	0.030	0.001
AVE			0.672		0.005

*p < 0.05, **p < 0.01.

##### Data analysis and results

The data analysis in this study employs Partial Least Squares (PLS), which is less restrictive with respect to sample size requirements and less sensitive to non-normal distributions, making it well suited to the analytical context of this study (Chin et al., 2003).

We first use Confirmatory Factor Analysis (CFA) to test the validity of the model. As shown in Table 3, all factor loadings exceed the 0.7 threshold (0.710 ~ 0.930), demonstrating good reliability; the Cronbach’s alpha fluctuates between 0.737 and 0.904, indicating good internal consistency. Additionally, the Composite Reliability (CR) values range from 0.835 to 0.940, demonstrating excellent internal consistency.

**Table 3:** Items’ loadings, reliabilities, and AVEs.

Item	Loadings	Alpha	CR	AVE
EP1	0.775	0.769	0.852	0.591
EP2	0.732			
EP3	0.780			
EP4	0.786			
NP1	0.883	0.823	0.894	0.738
NP2	0.838			
NP3	0.856			
IE1	0.828	0.828	0.886	0.660
IE2	0.785			
IE3	0.801			
IE4	0.834			
TP1	0.907	0.904	0.940	0.838
TP2	0.910			
TP3	0.930			
Acc1	0.837	0.741	0.853	0.659
Acc2	0.765			
Acc3	0.831			
Imm1	0.760	0.737	0.835	0.559
Imm2	0.759			
Imm3	0.759			
Imm4	0.710			
IB1	0.876	0.808	0.886	0.722
IB2	0.819			
IB3	0.853			

EP, Emotional Persuasion; NP, Narrative Presence; IE, Interactive Engagement; TP, Time Pressure; Acc, Accuracy; Imm, Immersion; IBB, Impulsive Buying.

Next, we examined convergent validity and discriminant validity. The former tested the Average Variance Extracted (AVE), which can effectively explain the ratio between the explained variance and the measurement error. As shown in Table 2, AVE values ranged from 0.559 to 0.838. The judgment of discriminant validity is mainly based on the diagonal elements of all constructs (the square root of AVE) (Fornll and Larcker, 1981). As shown in Table 4, all values exceed the corresponding diagonal values, indicating good discriminant validity.

**Table 4:** Constructs’ correlations and the squared root of AVEs (on diagonal).

	(1) Narrative Presence	(2) Immersion	(3) Impulsive buying	(4) Emotional persuasion	(5) Time pressure	(6) Accuracy	(7) Interactive engagement
1. Narrative presence	0.859						
2. Immersion	0.655	0.747					
3. Impulsive buying	0.420	0.488	0.850				
4. Emotional persuasion	0.603	0.587	0.455	0.768			
5. Time pressure	−0.117	−0.195	−0.056	−0.240	0.916		
6. Accuracy	0.509	0.601	0.366	0.523	−0.284	0.812	
7. Interactive engagement	0.527	0.499	0.422	0.404	−0.140	0.446	0.812

**Figure 2:**
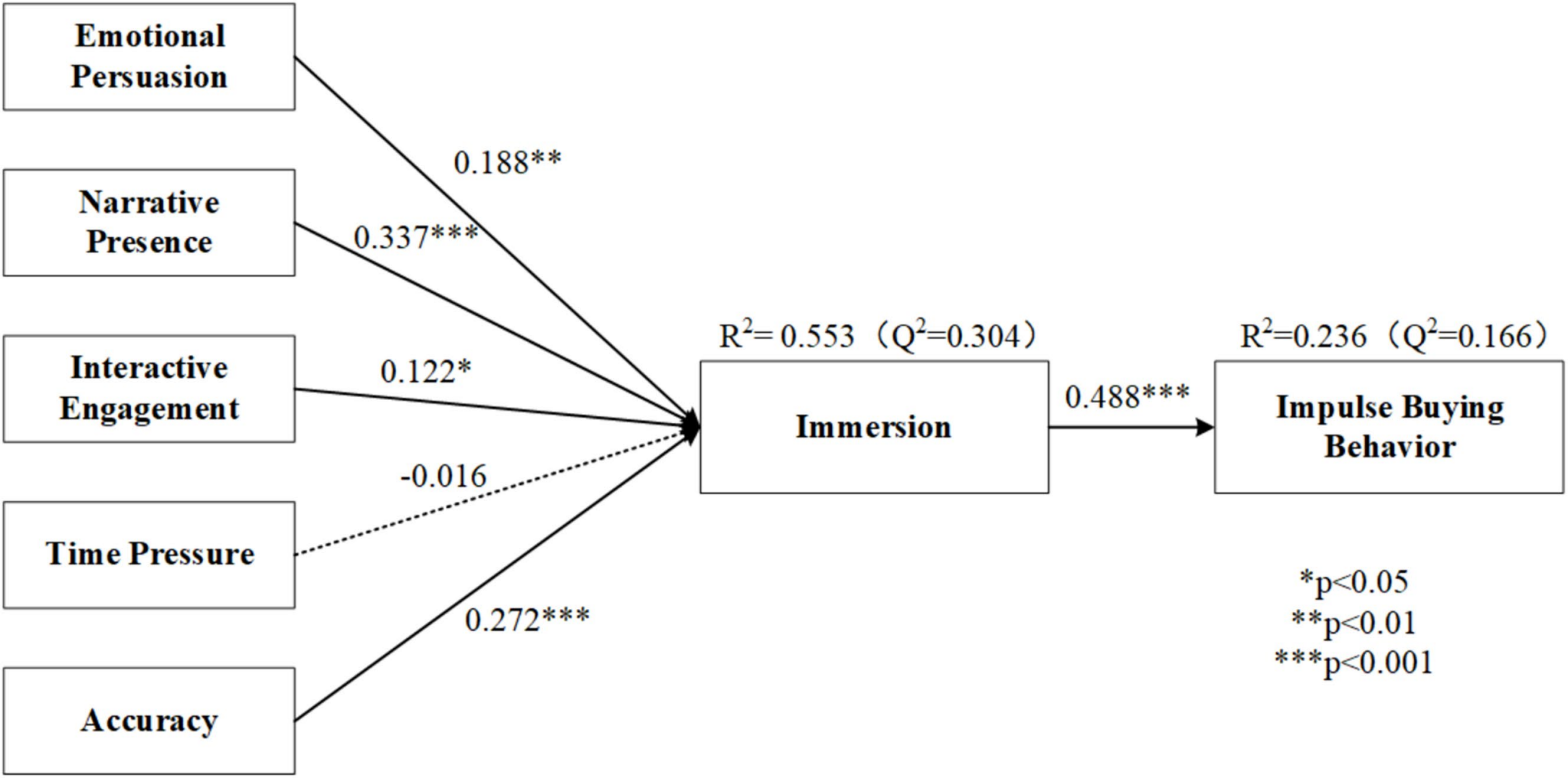
Results of the model.

The SEM results can be found in Figure 2, with the adjusted R-squared for immersion being 55.3% and the adjusted R-squared for impulse buying being 23.6%. All VIF values (1.318–3.453) were below the conservative threshold of 5, indicating that multicollinearity was not a concern in the structural model. In addition, effect size analysis indicates that immersion has a medium effect on impulsive buying behavior (*f*^2^ = 0.312). The model also demonstrates satisfactory predictive relevance, with *Q*^2^ values of 0.304 for immersion and 0.166 for impulsive buying behavior.

The structural model results demonstrated that most of the proposed hypotheses were supported. Specifically, emotional persuasion (H1), narrative presence (H2), interactive engagement (H3), and accuracy (H5) all showed significant positive effects on immersion, highlighting their roles as key factors associated with immersive experiences in live-stream shopping scenarios. Among these, narrative presence and accuracy exhibited particularly strong effects. In contrast, time pressure (H4) did not show a significant impact on immersion, indicating that urgency cues may not necessarily enhance users’ psychological engagement in this context. Finally, immersion (H6) had a significant positive effect on impulsive buying behavior, reinforcing its central role in stimulating spontaneous purchase decisions. These findings suggest that immersive psychological states are primarily shaped by affective and experiential cues rather than situational stressors. Moreover, immersion serves as a crucial psychological pathway driving impulsive consumption behavior in real-time commerce environments.

### FsQCA analysis

#### Calibration

In addition to SEM, this study employs fuzzy set qualitative comparative analysis (fsQCA) to complement the variable-centered, net-effect perspective of SEM. While SEM is used to examine the average linear relationships and mediation mechanisms among variables, fsQCA adopts a configurational approach to explore asymmetric relationships and identify multiple combinations of conditions that are sufficient for the outcome. Specifically, fsQCA addresses questions such as “What combinations of configurations of conditions are related to the expected outcomes?” (Fiss, 2007; Ragin, 2008). The QCA method can also be subdivided into crisp set qualitative comparative analysis (csQCA), multi-value set qualitative comparative analysis (mvQCA), and fuzzy set qualitative comparative analysis (fsQCA). Compared to the other two qualitative comparative analysis methods, the fsQCA method has advantages in handling continuous variables, making it more suitable for this study.

According to the steps of fsQCA analysis, before conducting the formal analysis, the original data needs to be calibrated. The data in this article is scale data. Based on existing theories and empirical knowledge, this study uses the direct calibration method to convert the data into fuzzy set membership scores, with the calibration standards for full membership, crossover point, and full non-membership set at 0.95, 0.5, and 0.05, respectively. Table 4 presents the calibration information for each condition and result in this article (Prentice et al., 2021; Woodside et al., 2014).

#### Necessity analysis

After the calibration is completed, a necessity analysis is conducted for individual condition variables. In fsQCA, if a condition is always present when the outcome occurs, then that condition is a necessary condition and should be eliminated. The consistency level is usually used to measure necessary conditions; if the consistency level is greater than 0.9, then that condition is considered a necessary condition (He et al., 2024; Schneider, 2012).

Table 4 shows the results of the necessity test for impulsive buying, where the consistency levels of all condition variables are less than 0.9, indicating that there are no necessary conditions, and further analysis can be conducted.

#### Truth table and solution analysis

We constructed a truth table to identify the configurations that influence impulse buying and explored the final solution based on frequency and consistency criteria. Configurations with a frequency threshold below 3 and a consistency value below 0.85 were disregarded (Al-Emran et al., 2024; Fiss, 2011; Greckhamer et al., 2013).

**Figure 3:**
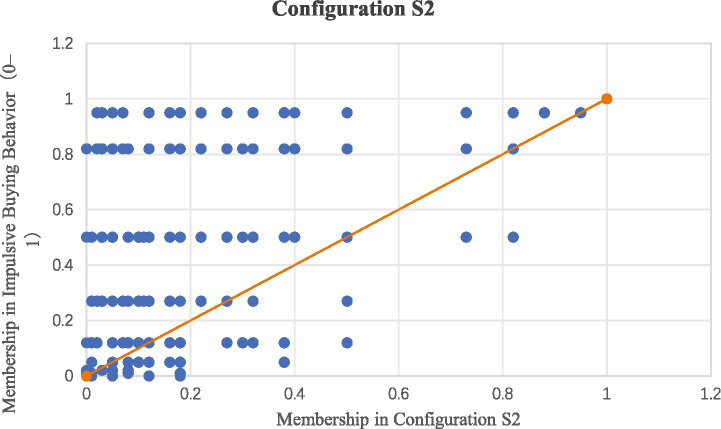
Scatterplot of configuration S2 for high impulsive buying behavior.

The results presented in Table 5 reveal eight sufficient configurations for high impulsive buying behavior. Configuration 1: When emotion persuasion, interactive engagement, accuracy, and immersion are partially present, impulsive buying behavior is significantly elevated. Configuration 2: Impulsive buying behavior is enhanced when emotion persuasion, narrative presence, accuracy and immersion are partially present. Configuration 3: When emotion persuasion, interactive engagement and immersion are fully present, narrative presence is partially present, impulsive buying behavior increases. Configuration 4: High impulsive buying behavior is observed when narrative presence and immersion are fully present, along with time pressure being partially present, while interactive engagement is fully absent and accuracy is partially absent. Configuration 5: Impulsive buying behavior is elevated when emotion persuasion, narrative presence and interactive engagement are fully absent, along with immersion being partially absent, and both time pressure and accuracy are fully present. To improve the interpretability of the configurational results, Figure 3 presents a scatterplot illustrating the sufficiency of Configuration S2 for high impulsive buying behavior, showing that most cases with high membership in Configuration S2 also exhibit high membership in impulsive buying behavior.

**Table 5:** Calibration and FSQCA results for high impulsive buying behavior.

	Solutions							
Configuration	Calibrations (0.95, 0.5, 0.05)	Consistency	Coverage	Solution 1	Solution 2	Solution 3	Solution 4	Solution 5
Emotion persuasion	(4.75, 4.25, 2.75)	0.797948	0.769801	●	●	●		⊗
Narrative presence	(4.6667, 4.3333, 2.33333)	0.738672	0.810105		●	●	●	⊗
Interactive engagement	(4.75, 4.5, 3.5)	0.668538	0.820514	●		●	⊗	⊗
Time pressure	(5, 4, 1.61667)	0.651917	0.680823				●	●
Accuracy	(4.6667, 4.3333, 2.61667)	0.723331	0.807499	●	●		⊗	●
Immersion	(4.75, 4.25, 3.25)	0.808577	0.754254	●	●	●	●	⊗
Raw coverage				0.540523	0.571761	0.553653	0.31682	0.201005
Unique coverage				0.0233157	0.0282798	0.0197817	0.0235168	0.0375676
Consistency				0.937714	0.92271	0.941273	0.931222	0.859627
Solution coverage				0.705976				
Solution consistency				0.889816				

A large filled circle (●) indicates the presence of a core condition, and a small filled circle (●) indicates the presence of a peripheral condition.

A large crossed circle (⊗) indicates the absence of a core condition, and a small crossed circle (⊗) indicates the absence of a peripheral condition.

Based on the results of the two research methods, both the fsQCA analysis and the SEM path model analysis emphasize the importance of emotional persuasion, narrative presence, and immersion, while also highlighting the complementary insights into how these factors interact and influence consumer behavior.

## Discussion

### Emotion persuasion and narrative presence as key mechanisms

Emotional persuasion (*β* = 0.188, *p* < 0.01) and narrative presence (*β* = 0.337, *p* < 0.001) have significant direct effects on immersion, as revealed by the SEM results. However, the fsQCA configurations demonstrate the flexibility of these two variables in different contexts. For example, in Configuration 1, emotional persuasion serves as a peripheral condition that, in combination with accuracy, interactive engagement, and immersion, promotes impulsive buying behavior. Meanwhile, in Configurations 2 and 4, narrative presence plays a more dominant role, functioning as a core condition that works alongside immersion to influence consumer purchase intentions. These findings indicate that while emotional persuasion and narrative presence have distinct focuses, they complement each other in influencing consumers’ psychological mechanisms and behaviors.

This conclusion aligns with prior research, emotional persuasion mainly triggers consumers’ emotional responses through strategies such as enthusiastic tone and emotional resonance, building a psychological connection on an emotional level (Li et al., 2024). On the other hand, narrative presence uses storytelling and context-based content design to create a concrete and perceivable consumption scenario for consumers, thereby enhancing immersion and fostering a sense of identification (Wang et al., 2022). This complementary effect is particularly evident in Configuration 3. In this configuration, emotional persuasion and narrative presence act as core and peripheral conditions, respectively, and jointly promote impulsive buying behavior through immersion.

### Context-dependent role of interactive engagement

SEM analysis reveals that interactive engagement has a significant but relatively weak positive effect on immersion (*β* = 0.122, *p* < 0.05), aligning with prior research conclusions (Gu et al., 2023; Wu et al., 2023). The fsQCA results further highlight the flexibility of interactive engagement in different combinations of conditions. For instance, in Configurations 1 and 3, interactive engagement, emotional persuasion, and immersion are identified as core factors related to impulsive buying behavior. However, in Configurations 4 and 5, interactive engagement is either entirely absent or plays a marginal role, suggesting that its influence is context-dependent. This implies that interactive engagement is more critical in scenarios where real-time connections between consumers and hosts need to be enhanced. In contrast, its importance may diminish when narrative and emotional elements are already strong.

This emphasizes the need for strategies to enhance interactivity based on specific contexts. For example, in scenarios where emotional and narrative elements are insufficient, real-time Q&A, consumer polling, or interactive comments can be used to bridge the gap and strengthen the emotional connection with consumers.

### Supporting roles of time pressure and accuracy

The time pressure factor has shown a secondary supplementary role in the results of both fsQCA and SEM. Specifically, time pressure exhibits contextual effects in the fsQCA analysis. For instance, the effects of time pressure are more pronounced and concentrated under the conditions of configurations 4 and 5. However, the SEM analysis indicates that time pressure, as a standalone condition, does not show a direct impact on immersion (*β* = −0.016, n.s) or impulsive buying behavior. This is consistent with the overall observation from fsQCA that time pressure is not a primary driving factor for impulsive buying and lacks universality. This finding contradicts previous studies on the effects of time pressure (Liu et al., 2022; Zhang, 2023), but aligns with narrative transportation theory. According to this theory, when individuals are deeply immersed in the narrative transportation process, their attention focuses on the story’s context, making external distractions (such as time or space) less relevant or even negligible (Escalas, 2004; Green and Brock, 2000; Green and Clark, 2013). This suggests that in the highly immersive and fast-paced virtual environment of shopping live broadcasts, the presence of strong narratives and emotional engagement reduces the impact of time pressure, making its effects more context-specific or conditional.

Similarly, accuracy demonstrates a certain level of importance in both SEM and fsQCA results (SEM: *β* = 0.272, *p* < 0.001; fsQCA: a supporting condition in Configurations 1 and 2). Clear and accurate information enhances consumers’ trust in the product, especially in contexts where interactivity is weak or emotional connection is lacking, serving as a compensatory factor.

### Immersion as a central mechanism

Both SEM and fsQCA results confirm that immersion is a key psychological mechanism driving impulsive buying behavior (SEM: *β* = 0.488, *p* < 0.001; fsQCA: a core condition in multiple configurations). SEM analysis shows that immersion is significantly influenced by emotional persuasion, narrative presence, and interactive engagement, as well as by accuracy (*β* = 0.272, *p* < 0.001). Meanwhile, the fsQCA results demonstrate that immersion consistently serves as a core condition across multiple configurations (Configurations 1, 2, 3, and 4). In these configurations, immersion either operates in combination with emotional persuasion and interactive engagement or functions as a complementary condition alongside narrative presence and time pressure.

These findings highlight the critical role of immersion as a “bridge” that connects antecedent variables to impulsive buying behavior. When consumers are immersed in the live-stream shopping context, their attention becomes focused on the host’s presentation and product demonstration. This heightened attentional focus makes them more susceptible to emotional and psychological influences, which may in turn lead to immediate purchase decisions (Gu et al., 2023; Joo and Yang, 2023).

### The necessity of multi-factor combinations

The diverse configurations identified in the fsQCA analysis highlight the multifactorial nature of impulsive buying behavior, where different combinations of core and peripheral conditions are associated with the outcome. This aligns with the SEM finding that no single factor is sufficient to fully explain consumer behavior. Instead, factors like emotion persuasion and narrative presence must work in tandem with supporting elements like accuracy and interactive engagement to create a compelling consumer experience. This multi-factor perspective underscores the necessity of combining the linear, single-factor focus of SEM with the configurational, holistic approach of fsQCA to provide a more nuanced understanding of consumer behavior.

## Conclusion

### Theoretical contributions

This study extends narrative transportation theory from communication and advertising into digital consumption contexts, emphasizing its interdisciplinary relevance for consumer behavior. It offers a theoretical lens to explain how narrative features, particularly **e**motional persuasion and narrative presence, shape audience immersion and influence purchasing decisions (Green and Brock, 2000; Green and Clark, 2013).

Unlike prior studies focusing on hosts’ external traits, this research systematically deconstructs the narrative content of live-streaming hosts and highlights its central role in influencing consumer psychology and behavior. Emotion persuasion and narrative presence emerge as key factors in driving immersion and purchase intention, while factors like time pressure appear more context-dependent. This shift from host persona to message-level analysis contributes novel insights to live-stream shopping research.

Methodologically, the study applies a mixed-methods design, integrating qualitative content analysis with SEM and fsQCA. SEM validates the causal effects and relative strength of each content feature, while fsQCA reveals diverse configurations of influencing factors, such as the synergy between narrative presence and emotional persuasion. This dual approach addresses limitations of single-method research and provides a richer understanding of nonlinear interactions in consumer decision-making. Overall, the study contributes both theoretical depth and methodological advancement, offering a valuable reference for future interdisciplinary and multi-variable consumer research.

### Practical implications

First, the findings provide important guidance for live-stream hosts in improving their narration strategies. Hosts can enhance emotion persuasion by employing an enthusiastic tone, fostering a positive emotional atmosphere, and creating resonance with their audience. Additionally, narrative presence can significantly increase emotional engagement and immersion. This is achieved through practices such as delivering brand stories or showcasing product usage scenarios that resonate with consumers’ everyday lives. These strategies help establish emotional connections between hosts and consumers, boosting trust in the product and increasing purchase intentions. Moreover, hosts should tailor their narration content to meet the needs and preferences of their target audience, incorporating cultural relevance or trending topics to make their presentations more personalized and appealing. At the same time, maintaining authenticity and consistency in their messaging is crucial for building trust and fostering long-term consumer relationships and brand loyalty.

Second, this study offers practical insights for brand owners to support and optimize live-stream narration strategies. Brand owners should actively assist hosts in improving the quality of their content, particularly by providing support in narrative design and product presentation. For example, supplying detailed and compelling brand stories or real-world product use cases can help hosts effectively communicate product value to consumers. Additionally, brand owners must ensure the clarity and accuracy of product information shared during the live stream to build consumer trust. Besides, brands can design more interactive activities with hosts, such as interactive Q&A with consumers, allowing consumers to participate in voting for product selection, etc. Such activities can enhance consumers’ sense of participation and interaction conversion rate, not only improving the effectiveness of the host’s narrative but also strengthening the social connection with consumers.

### Limitations and future work

Several limitations should be acknowledged. First, the generalizability of the findings may be constrained by the research context and sample characteristics. The data were collected within Chinese live-streaming commerce, where live shopping is highly institutionalized, and consumer responses to host narration strategies may differ in other cultural settings. In addition, the sample is skewed toward younger consumers, which reflects the dominant live-streaming audience but may limit applicability to older age groups.

Second, this study does not explicitly differentiate between product categories. Narrative strategies may function differently across utilitarian versus hedonic products or across varying levels of purchase involvement, which warrants further investigation.

Finally, this study adopts a cross-sectional design, limiting insights into how consumer responses evolve over time. Future research could employ longitudinal or experimental designs to examine whether immersion and impulse buying responses change with repeated exposure or prolonged engagement.

## Data Availability

The raw data supporting the conclusions of this article will be made available by the authors, without undue reservation.

## Appendix A

**Table:**

Construct	Item	Measurement Item (English translation)
**Emotional Persuasion** (Walters et al., 2010)	EP1	Live-streaming host’s narration is very appealing to me.
	EP2	Live-streaming host’s narration makes me feel good.
	EP3	Live-streaming host’s narration arouses my interest in watching.
	EP4	Live-streaming host’s narration stimulates my emotions.
**Narrative Presence** (Busselle and Bilandzic, 2009)	NP1	At times during the live-streaming, I felt as if I were inside the scene created by the host’s narration.
	NP2	During the live-streaming, my attention felt focused on the narrated environment rather than on my physical surroundings.
	NP3	After the live-streaming ended, the narrated world seemed to disappear, and I returned to the real world.
**Interactive Engagement** (Yoo et al., 2010)	IE1	The live-streaming host pays attention to viewers’ comments and feedback during the session.
	IE2	The live-streaming host facilitates two-way communication with viewers.
	IE3	The live-streaming host listens to viewers’ opinions and feedback.
	IE4	The live-streaming host provides viewers with convenient opportunities to respond and give feedback.
**Time Pressure** (Gärling et al., 2015)	TP1	During the live-streaming, the host frequently emphasizes that time is limited.
	TP2	During the live-streaming, I frequently feel urged to make a purchase.
	TP3	During the live-streaming, I frequently feel rushed due to insufficient time to place an order.
**Accuracy** (Wixom and Todd, 2005)	Acc1	Overall, the information provided by the live-streaming host is correct.
	Acc2	There are few errors in the basic attributes and data-related information I obtain from the live-streaming host’s narration.
	Acc3	The information provided by the live-streaming host is accurate.
**Immersion** (Gong et al., 2024)	Imm1	The interaction between the live-streaming host and viewers feels close and engaging.
	Imm2	I feel comfortable interacting with the live-streaming host and other viewers during the live-streaming.
	Imm3	I am willing to engage in interactions with the host (or other viewers) during the live-streaming.
	Imm4	I am willing to actively communicate with the host during the live-streaming.
**Impulsive Buying** (Fu and Hsu, 2023)	IB1	During the live-streaming, I suddenly felt an urge to purchase items beyond my original shopping plan.
	IB2	During the live-streaming, I felt a desire to buy products that were not part of my original shopping plan.
	IB3	During the live-streaming, I had thoughts or inclinations to purchase items outside my planned purchases.
